# Society Meetings

**Published:** 1907-02

**Authors:** 


### SOCIETY MEETINGS.

### The State Medical Society.

THE one hundred and first annual meeting of the Medical Society of the State of New York was held at Albany on January 29 and 30, 1907. The meeting was one of the most successful in recent years and the following program of the scientific session is one of unusual excellence:

Tuesday, January 29, 1907.

President's address.
Reception of delegates from other societies.

Danger Signals from the Skin—L. Duncan Bulkley, New York.

The Importance to Healthy Individuals of Periodic Aural Examination with Functional Tests—W. Sohier Bryant, New York.

Practical Legislation for the Prevention of Blindness from Ophthalmia Neonatorum—F. Park Lewis, Buffalo.

A Plea for New Methods in the Prevention of Blindness—Lucien Howe, Buffalo.

Chloroma, with Special Reference to the Ocular Symptoms—C. S. Merrill and A. J. Bedell, Albany.

SYMPOSIUM ON NON-TUBERCULOUS JOINT INFECTIONS.

1. The Pathology of Non-tuberculous Joint Infections with exhibition of specimens—E. H. Nichols, Boston.
2. Pneumococcus and Typhoid Infections—Roswell Park, Buffalo.
3. Syphilitic and Gonorrheal Infections—R. H. Sayre, New York.
4. Staphylococcus and Streptococcus Infections—Lucius W. Hotchkiss, New York.

5. Diagnosis and Symptoms of the Rheumatoid Diseases—R. R. Fitch, Rochester.

6. Mechanical Treatment—H. L. Taylor, New York.

7. Operative Treatment. Walter Wood, Brooklyn.

New York State Medical Library—Albert Vander Veer, Albany.

State Aid for Medical Libraries—Smith Boker, Utica.

Some Questions Involved in Sending an Invalid Away from Home—J. H. Pryor, Buffalo.

Sanitorium Treatments; their Therapeutic Value—P. O. Kinnear, Clifton Springs.

Syphilitic Lesions of the Eyelids, with Reports of Cases—Frank Judson Parker, New York.

Some of the Fallacies in the Treatment of Syphilis—Samuel Alexander, New York.

The Spirocheta Pallida with Lantern Slide Demonstration—James Ewing, New York.

SYMPOSIUM ON CANCER.

A Synopsis of the Work of the Buffalo State Laboratory during the past Eight Years. Retrospective and Prospective—Roswell Park, Buffalo.

Parasitism and Infection—Harvey R. Gaylord, Buffalo.

Cancer and Immunity—G. H. A. Clowes, Buffalo.

Cancer as a Biological Problem—Gary N. Calkins—New York.

Lantern Slide Demonstration and Exhibition of Specimens.

WEDNESDAY, JANUARY 30, 1907.

Sahli's Desmoid Reaction—H. W. Carey, Troy.

The Abortive Treatment of Pneumonia—G. Lenox Curtis, New York.

A New Disease: History, Symptoms and Pathology of a Hitherto Unreported Lesion—Henry P. De Forest, New York.

Blood Pressure Study; Some Unexpected Revelations—Henry L. Elsner, Syracuse.

The Clinical Forms of Blood Pressure Changes—Louis Faugéres Bishop, New York.

Some Clinical Experiences with Arteriosclerosis—Henry G. Webster, Brooklyn.

The Criminal Lunatic; His Status and Disposition—Robert B. Lamb, Matteawan.

Imperative Impulses in the Neurotic Resembling Obsessions in the Insane—Edward D. Fisher, New York.

Visions of Mary Czajka—Francis E. Fronczak, Buffalo.

Typhoid Fever; Report of a Series of Cases with a Low Death Rate—William S. Hubbard, Brooklyn.

The Importance of the Routine Examination of the Urine for Indican—Joseph Day Olin, Watertown.

Underfeeding and its Associated Ills—Dudley D. Roberts, Brooklyn.

A Certain Danger in the Use of Chloroform—Arthur W. Booth, Elmira.

The Technic of the Ablation of the Breast—Parker Syms, New York.

Surgical Treatment of Goiter—Martin B. Tinker, Ithaca.

Clinical Features and Operative Treatment of Thyroid Affections—George E. Beilby, Albany.

Some Recent Clinical Observations in Intestinal Obstruction, both Acute and Chronic, with Demonstration of Specimens and Illustrations—Joseph C. Bloodgood, Baltimore.

The Surgery of Foreign Bodies in the Respiratory Tract—W. G. Macdonald, Albany.

Contribution to the Casuistry of Foreign Bodies in the Uterus—B. S. Talmey, New York.

Further Observations Upon the Treatment of Diffuse Septic Peritonitis, with a report of One Hundred and Forty-five Cases Treated by the Elevated Head and Trunk Position—Russell S. Fowler, Brooklyn.

Cyst of the Mesentery; Resection of Twenty Inches of Small Intestine; Recovery—Edgar A. Vander Veer, Albany.

Mechanical (Surgical) Constipation—Samuel G. Gant, New York.

The Treatment of Ventral Hernia—A. V. Moschowitz, New York.

Vaginal Cesarean Sections, their Limitations, Indications and Technic, with Report of Two Cases—H. Judson Lipes, Albany.

The annual banquet was held at the Hotel Ten Eyck on January 30, at 7:30 P. M. The officers elected were—president. Frederick C. Curtis, Albany; vice-presidents, Julius Bierwirth, Brooklyn, Edward Torrey, Allegany, and Nelson G. Richmond, Fredonia; secretary, Wisner R. Townsend, New York; treasurer, Alexander Lambert, New York.

---

The Buffalo Academy of Medicine held meetings during the month of January, 1907, as follows:

At a stated meeting of the Academy, January 8, a discussion for improving and increasing the city water supply was held. The various proposed measures were discussed by Col Francis G. Ward, commissioner of public works; Mr.

George R. Sikes, civil engineer; Mr. Frank J. Tresise, civil engineer; councilman John J. Smith, and Dr. P. W. Van Peyma.

*Section on Medicine.*—Tuesday, January 15. Program: symposium on gastric ulcer: (a) medical aspects, Albert G. Woehnert; (b) surgical aspects, Eugene A. Smith; (c) discussion by Charles G. Stockton, Marshall Clinton.

*Section on Obstetrics and Gynecology.*—Tuesday, January 29. Program: (a) prolapse of the uterus, Charles E. Congdon; (b) unnecessary operations, especially in the female, J. Henry Dowd; (c) Herman E. Hayd exhibited a specimen of lithopedion, of 32½ years' duration, which he removed from a woman 67 years of age.

---

At the annual meeting of the Harvard Medical Society of New York, held at the residence of Dr. George E. Brewer on January 26, 1907, the following officers were elected for the ensuing year: president, Dr. J. Hilton Waterman; vice-president, Dr. Carleton P. Flint; secretary, Dr. George D. Scott; treasurer, Dr. Edward W. Pinkham; executive committee, Drs. W. Sohier Bryant, Edward K. Dunham, Follen Cabot.

---

The regular meeting of the Esculapian Club was held at the Touraine Hotel, Thursday, January 17, at 8:30 P. M. The host was Dr. J. W. Charters and the subject of the evening's discussion was "Bradycardia," with presentation of cases.

---

At the annual meeting of the Allegany Society, of Buffalo, held on January 10, 1907, Dr. Albert E. Persons was elected president.

